# An introductory review of post-resection chemotherapeutics for primary brain tumors

**DOI:** 10.37349/etat.2023.00150

**Published:** 2023-06-30

**Authors:** Meaghan McGovern, Michaela Scanlon, Amanda Stanton, Brandon Lucke-Wold

**Affiliations:** Università degli studi della Campania “Luigi Vanvitelli”, Italy; ^1^School of Medicine, Uniformed Services University of the Health Sciences, Bethesda, MD 20814, USA; ^2^Department of Neurosurgery, University of Florida, Gainesville, FL 32608, USA

**Keywords:** Chemotherapeutics, primary central nervous system tumor, blood brain barrier

## Abstract

The treatment of central nervous system (CNS) tumors is complicated by high rates of recurrence and treatment resistance that contribute to high morbidity and mortality (Nat Rev Neurol. 2022;18:221–36. doi: 10.1038/s41582-022-00621-0). One of the challenges of treating these tumors is the limited permeability of the blood brain barrier (BBB). Early pharmacologic treatments worked to overcome the BBB by targeting vulnerabilities in the tumor cell replication process directly through alkylating agents like temozolomide. However, as advancements have been made options have expanded to include immunologic targets through the use of monoclonal antibodies. In the future, treatment will likely continue to focus on the use of immunotherapies, as well as emerging technology like the use of low-intensity focused ultrasound (LIFU). Ultimately, this paper serves as an introductory overview of current therapeutic options for post-resection primary brain tumors, as well as a look towards future work and emerging treatment options.

## Introduction

While treatment advancements in neurosurgery, chemotherapy, and radiation for primary central nervous system (CNS) tumors have improved, there are still high rates of recurrence and treatment resistance [1]. The challenge in treating these tumors is multifactorial and arises from difficulty achieving full tumor resection, poor penetration of the blood brain barrier (BBB) by chemotherapy, and intrinsic treatment resistance bred within the tumor microenvironment. This paper serves as a literature review of the current standard of care for post-surgical resection chemotherapy of CNS tumors, as well as an introduction to emerging pre-clinical drugs that may improve the management of primary CNS tumors.

Primary CNS tumors are organized into glial and non-glial cell tumors. The glial tumors include astrocytoma, oligodendroglioma, and ependymoma. Glioblastoma multiforme (GBM), a subtype of astrocytoma, is the most common and aggressive malignant tumor found in the adult CNS [2]. Non-glial cell tumors include meningiomas, schwannomas, craniopharyngiomas, and pituitary gland tumors. The fifth edition of the World Health Organization (WHO) classification of Tumors of the CNS is the guiding body of literature by which these tumors are graded [3]. This most recent edition introduced the major advancements in molecular diagnostics that now guide tumor classification and treatment.

## Alkylating agents

Currently, the most common chemotherapeutics used for post-resection brain tumors are alkylating agents. The path to the creation of alkylating agents began when it was discovered that chemicals being used as military weapons caused bone marrow suppression and lymphoid aplasia [4]. Innovation in the field of alkylating agents has since led to the discovery of applications for brain tumor management. The mechanism of actions of current alkylating chemotherapeutic medications approved by the Food and Drug Administration (FDA) for brain tumors are outlined in Table 1 [5].

**Table 1:** FDA-approved alkylating agents for the treatment of brain tumors

**Medication**	**Tumor type**	**Mechanism of action**	**Reference**
Temozolomide	GBM and anaplastic astrocytoma	Alkylating agent that leads to methylation and later apoptosis of impacted cells	[6]
Carmustine	Glial tumors (wafers are frequently placed at the time of GBM resection as a bridge to radiation therapy)	Alkylating agent administered via intravenous injection or local implantation in wafer form	[7]
Lomustine	Glial and non-glial cell tumors like medulloblastoma	Nitrosourea works as an alkylating agent	[8, 9]

Temozolomide is the most commonly used alkylating agent. One of its current applications is as an adjunct to radiotherapy for post-resection GBM, known as the Stupp protocol [10]. The use of temozolomide in combination with surgery and radiotherapy still only remains at a median progression-free survival of 6.7 months, and a median survival time of 16.6 months [11].

## Monoclonal antibodies

Monoclonal antibodies are promising in the field of oncology to treat tumors outside the CNS as antibodies serve as tumor-specific treatments [12]. Initially, the BBB presented a barrier to the application of monoclonal antibodies to brain tumor treatment [12]. For example, in breast cancer, which is responsive to monoclonal antibodies like trastuzumab, metastasizes to the brain have been observed even in the presence of successful local treatment [12]. However, further research into the field demonstrated treatment benefits through the use of monoclonal antibodies such as Bevacizumab and Naxituamab, which are currently FDA-approved for the treatment of brain tumors. The mechanism of action and applications of current monoclonal antibodies for tumor management are summarized in Table 2.

**Table 2:** Monoclonal antibodies approved for the treatment of brain tumors

**Medication**	**Tumor type**	**Mechanism of action**
Bevacizumab	Glial tumors which include GBM (primary and recurrent) [13]	Binds to and inhibits VEGF leading to a reduction in microvascular growth which limits blood supply to tumors [14]
Naxitamab-gqgk	Neuroblastoma and GD-2 positive cancers [15]	Humanized monoclonal antibody [15, 16]
Dinutuximab [16]	Neuroblastoma	Anti-GD-2 monoclonal antibody [16]

VEGF: vascular endothelial growth factor

The path to applying monoclonal antibody use to brain tumor treatment was paved through an evaluation of how tumor-mediated damage to the BBB contributes to pathogenesis. High grade gliomas, such as glioblastoma, have high amounts of VEGF. The high expression of VEGF leads to a highly vascularized, disorganized, and leaky BBB. As the BBB serves as a protective mechanism for the brain, the damage to the structure that results from high levels of VEGF leads to vasogenic edema [13]. This suggests that VEGF may be a potential target for the treatment of brain tumors.

The first study evaluating the preclinical efficacy of an anti-VEGF agent for brain tumors was published in 2000 by Rubenstein et al. [17]. It demonstrated slowed growth of tumors and therefore the potential for prolonged survival. However, their model also demonstrated concern for tumor mutations leading to angiogenesis and satellite tumors [17]. More work in pre-clinical and clinical applications of anti-VEGF antibodies was needed in order to determine the utility in brain tumor treatment.

Bevacizumab, a monoclonal antibody that inhibits VEGF, demonstrated success in the treatment of other highly vascular tumors such as colorectal [18]. Thus, it was hypothesized that similar success could be seen in high grade gliomas. Early phase II trials of the use of bevacizumab as a single agent, as well as a combination agent, were successful and demonstrated increased progression free survival times [18]. In 2009 Bevacizumab received accelerated FDA approval when it was demonstrated to have a treatment benefit as a single-agent in the treatment of recurrent glioblastoma [13].

Naxitamab-gqgk is a monoclonal antibody that targets disialoganglioside GD-2 [19]. It was granted orphan designation by the FDA in 2013 and has been approved since 2020 in a combination therapy with sargramostim, a recombinant granulocyte-macrophage colony stimulating factor [19]. The combination therapy is approved for use in recurrent and refractory pediatric and adult high-risk neuroblastoma [19]. The function of disialonganglioside GD-2 in normal cells is to aid in cell communication and the attachment of cells [20]. Therefore, naxitamab seeks to disrupt this function and thereby induce cytotoxicity and cell death [16]. Naxitamab represents a second example of how the ability of immunotherapies for brain tumor treatment to capitalize on vulnerabilities increases their ability to be efficacious. Other anti-GD-2 immunotherapies include dinutuximab [16].

Dinutiximab differs from naxitamab because dinutuximab has a murine protein. Murine antibodies are less favored as they may trigger stronger immunologic responses and lead to allergic reactions. They also are known to lead to the development of human anti-mouse antibodies (HAMA). This feature of murine antibodies leads to an accelerated clearance of the antibody and less therapeutic impact on tumors [16]. Several other treatment options approved for the treatment of brain-tumors that are approved by the FDA are summarized in Table 3.

**Table 3:** Additional FDA-approved medication for brain tumor treatment

**Medication**	**Tumor type**	**Mechanism of action**
Evorlimus	Subependymal giant cell astrocytoma (associated with tuberous sclerosis), potential applications to pediatric tumors to include DIPG, peripheral primitive neuroectodermal tumors, anaplastic astrocytomas, and ependymomas [21]	Immunosuppressive macrolide that works in T-cells in response to alloantigen to stop growth-driven transduction signals [22]
Belzutifan	VHL associated tumors like hemangioblastoma [23]	HIF-2α inhibitor thus decreasing tumorigenesis [24]

DIPG: diffuse intrinsic pontine gliomas; VHL: Von Hippel-Lindau; HIF-2α: hypoxia-inducible factor-2alpha

## Combined therapies

Currently, the mainstay of FDA-approved combination chemotherapy options for the treatment of brain tumors is composed of procarbazine hydrochloride, lomustine, and vincristine sulfate (PCV). The mechanism of action of each component is outlined in Table 4.

**Table 4:** Mechanisms of action for components of PCV

**Medication**	**Mechanism of action**
Procarbazine hydrochloride	Possible mechanism is through the inhibition of trans-methylation of methionine into transfer-RNA (t-RNA) thus inhibiting protein synthesis [25]
Lomustine	Nitrosourea that works as an alkylating agent [9]
Vincristine sulfate	Inhibits the formation of microtubules by binding to tubulin which leads to mitosis arrest. It also is able to block glutamic acid utilization and thereby interfere with the synthesis of nucleic acids and proteins [26]

PCV was initially favored for the treatment of brain tumors such as anaplastic oligodendrogliomas prior to the introduction of temozolomide. However, since the introduction of temozolomide, the use of PCV has begun to fall out of favor due to the toxicity. The toxicities include severe weight loss, fatigue, neuropathy, and paralytic ileus [27]. The dose limiting toxicity for vincristine is damage to microtubule function in axons leading to neuropathy [28]. Despite the associated toxicities and difficulty of administration, retrospective studies of the use of PCV and radiotherapy suggest that PCV increases the time to progression (TTP) [29].

Data on PCV was measured in TTP. One study isolated PCV and temozolomide treatment in patients with 1p19q co-deletions as co-deleted anaplastic oligodendrogliomas are typically treated with chemotherapy alone. The study found that in cases with co-deletions PCV only treatment had a longer TTP of 7.6 years when compared to temozolomide alone [29].

In their study, Lassman et al. [29] note the known toxicity of PCV. In addition, they also raise the question of if increased TTP could leave patients at higher risk for toxicity sequelae like myelodysplasia [29]. In addition, it has been shown that while adjuvant PCV therapy increases progression free survival, it does not have a significant impact on overall survival [30]. Therefore, the use of combination radiation therapy with multiple chemotherapeutics has proven to be a balance between therapeutic benefit and toxicity.

## Emerging medications

Tyrosine kinase inhibitors are a type of targeted treatment that works by modifying the signals of tyrosine kinase [31]. The recent phase II relapsed glioblastoma (REGOMA) trial offers promising results regarding the use of the tyrosine kinase inhibitor regorafenib for the treatment of relapsed glioblastoma [32]. In the trial, regorafenib’s efficacy was compared to lomustine to evaluate for impacts on survival, as well as health-related quality of life. In both categories, regorafenib outperformed lomustine [33].

Options in pharmacologic intervention are further expanding with the emergence of molecular subclassifications and advancements in immunotherapy. Exploring the field of molecular markers allows scientists to better understand the tumor microenvironment, though this environment has proven to be diversely complex. Subtyping tumors based on the presence or absence of integral cell signaling molecules has led to advancements in targeted immunotherapy. A number of these therapies are being studied in the preclinical setting. For example, the ataxia-telangiectasia mutated (ATM) protein directs cell response to double-strand DNA breaks that are induced by radiation. Ablation or pharmacologic inhibition of this protein results in tumor cell hypersensitivity to radiation in the monkey brain [34]. An orally bioavailable ATM inhibitor, AZD1390, has been optimized to penetrate the BBB. It has been shown to radio-sensitize glioma cell lines with the tumor protein p53 (p53) mutant being more susceptible than wild-type. This medication, used in conjunction with radiotherapy, was shown to significantly induce tumor regression and increase animal survival when compared to radiotherapy alone [18].

An area of interest currently undergoing further study is the use of cytotoxic T lymphocytes (T cells). These cells can be extracted from a patient and modified to express a chimeric antigen receptor (CAR) specific to an identified tumor antigen [35]. This process leads to the specific identification and elimination of tumor cells, with relative sparing of non-tumor host cells compared to traditional chemotherapeutic agents. These genetically-modified T cells, known as CAR T cells, have proven to be successful in treating a number of hematologic cancers. As a result, this treatment modality is the focus of many research initiatives, to include the investigation of their efficacy in primary CNS tumors. Thus far, this avenue has been relatively unproductive in the way of CNS malignancies given the famously vast antigenic heterogeneity within the tumor microenvironment [36]. Additionally, solid tumors are typically foci of relative immunosuppression, which presents a challenge due to the limited efficacy of immunotherapy in these regions.

In addition, to work in chemotherapeutics there is also promising work being done outside of pharmacology in order to overcome the BBB. Recently, this barrier has been overcome with the utilization of LIFU in combination with intravenous- administered oscillating microbubbles (MBs) [37]. High-intensity focused ultrasound (HIFU) has been used to directly destroy glioblastoma tissue and its surroundings. In contrast, LIFU is a non-invasive technique where ultrasound waves converge, altering the permeability of the BBB and allowing drug delivery into a specific region of the brain [37]. These lower-energy, non-continuous waves focused on the exogenously administered MBs temporarily disrupt the barrier, allowing onco-therapeutics to cross over within the given time frame [38, 39]. The use of these lower modalities has been shown to be safe for the brain parenchyma.

## Conclusions

While the advancements in the treatment of primary CNS tumors are promising and have success in improving survival rates. They also show promise in applications beyond tumor treatment with potential applications to the treatment of Parkinsonian and Essential tremors. However, the limited treatment options also represent the relatively neoteric domain of tumor pharmacology. Moving towards the future, our understanding of the BBB will need to continue to be explored in order to better understand how to navigate its intricacies in order to more optimally and effectively treat patients.

## Abbreviation

BBB: blood brain barrier
CNS: central nervous system
FDA: Food and Drug Administration
GBM: glioblastoma multiforme
LIFU: low-intensity focused ultrasound
PCV: procarbazine hydrochloride, lomustine, and vincristine sulfate
TTP: time to progression
VEGF: vascular endothelial growth factor

## Declarations

### Copyright

© The Author(s) 2023.
